# Supplementary material for: C14ORF39/SIX6OS1 is a constituent of the synaptonemal complex and is essential for mouse fertility
Source: Nat Commun. 2016 Oct 31;7:13298. doi: 10.1038/ncomms13298 (PMC5095591; doi:10.1038/ncomms13298)
Supplement: Supplementary Information — Supplementary Figures 1-13, Supplementary Tables 1-2, Supplementary References. [file ncomms13298-s1.pdf]

Supplementary Information

```
L_chalumnae     -MKDHLLSNLDKFLLELVFQCEQASTCKEQEKQQIQIYITNVIEGKQEIGIHHENINQLDEEIVRLQKQNEQNKEKCK----TWKPTYVLFSEHEEHLQKELKSIQANNEKDKKAYQDQIIQ
O_anatinus      -MSDKLFINLNKFLLELVFQSERDSTAKEDVMQLTQICSAKVLESKKCILKLHENINKSDDEIVSYQKHKEHFKDSCI----NWKPTYDVLYKHENYLQEQFQKHQESTEKDKKMYHDYMSQ
G_gallus        MMSDRLLSNLDKLLLEFVFQLKQTSYAKEHVNQQINLYSSKITEKRNEIARLQENINNCNDAIADLRKQNENSKDSCN----AWKPTYAILSKHEEYLKNELEVLQEATENERKMYQDYIIQ
P_sinensis      -MNDTLLSNLDKLLLQLVFQLEQTSYAKEHVKQQINLYTASIVERKNEICRLHENINNSNDAIVYLHKYNKNNKDNCNVLGHSWKPTYVVLSKHEEYLNNQLKNCQETTESDKKMYQDYMNQ
S_harrisii      --MNDLFIALDKLLLELVFQSEQDSNAKAEAKQQIVLFYKNIKEKKKSIFSLHDKINAIDNEITHCRKHNKYVKENCA----NWMPTYDILHKHEVFLEDQFQNDQETTEKDKKVYHDQMRQ
M_musculus      -MNDNLFVSLDRLLLEFVFQYEQDISIKEDTIQRINKCLESIKENKANVSKLREAINKVDEDIAFHYKHSKEIKDSCS----NWKPTCDVFHKHEDYIKDQLTAYQETNEKDKKMYHDYICQ
P_troglodytes   -MNDSLFVSLDRLLLEFVFQYEQDISTKEEMIQRINKCCEDIKENKVTICRIHETINATDEEIDHYCKHSEEIKDNCR----NWKPTCDVFRKHEDYMQDQFTVYQETIEKDKEMYHDYICQ
H_sapiens       -MNDSLFVSLDRLLLEFVFQYEQDISTKEEMIQRINKCCEDIKENKVTICRIHETINATDEEIDHYCKHSEEIKDNCR----NWKPTCDVFRKHEDYMQDQFTVYQGTVEKDKEMYHDYICQ
                   : *:  *:::**::*** ::    *  .  *       .: * :  :   :: **  :: *    * .:  *:.*      * **  :: :** .::.::   *   *.::: *:* : *

L_chalumnae     CREILWKHQAQHSESSLAKEYFKKKASLDEIQTRVLKCTEEIKQKENIFMNLLEPAPFRSFHDWALQIV-SLRKNTQNTYKKALCLLNESSQLEKESLELER--NWLQKIS-----------
O_anatinus      HKKVFAQYQITYSERTLAKEYNRKKAEFEVIHNRVLSQTEKFKLKETTLMELLVPAPFRSLPHWALLIA-NLRYKTEDTLKQAASVTQKSLLLKKESEEMETKLRSLKNCFITLLVMKRISE
G_gallus        YKETLKQHREKYAESALAQEYYKKKKELEEIQNRIQKRSEQYKLKEDADLDILEPAPFKSINDWALYIA-SLRQKTQEMLKLAETATQESIELEKEADELEMKINYLKKTFEETTEDENNSE
P_sinensis      YKEIFKQHQAKYSENALAQEYYMKKKEIEEIRNTVLKHSEQFKWKEATLLAILEPAPFRSLSDWALQKSAYVRQKTQEVLKHAAVFTQKSIELVKETDEVEMKINYFKQQRERSTEDQDRSK
S_harrisii      CKDVLKHYQLQYSKAPLAQKYYKDKTQVEELKNQILACNEQCTIKETILLELSVPAPFKSLSQWALQIV-HLRIKTEDFLKCASTLIHKTCKMKEEIEEMER-----NQETERISEAKNLPE
M_musculus      YEDVLKQYQLKYSETRFSCKYYEKKKEHEEIKNRVLACTEQLQLNETILMKFLVPAPFPSLTKWTLYVV-NLRYRTQDILKRANNFTKRSFELEKEADDMEIEINSLNKM-ARLFESKTFSE
P_troglodytes   YKEVLKQYQLKYSETPFSREYYEKKREHEEIQSRVLACTEQLKMNETIFMKFRVPAPFPSLTKWTLNIV-NLRCETQDILKHASNLTKSSFELKKEVDEMEIEINYLNQQISRHNETKALSE
H_sapiens       YKEVLKQYQLKYSETPFSREYYEKKREHEEIQSRVLACTEQLKMNETIFMKFRVPAPFPSLTKWTLNIV-NLRCETQDILKHASNLTKSSSELKKEVDEMEIEINYLNQQISRHNETKALSE
                 .. : :::  :::  :: :*  .* . : ::. :   .*:   :*   : :  *****: .*:*      :* .*::  * *    . :  : :*  ::*      ::

L_chalumnae     --------------------FQK-----------------------------------------------------------------------TEAVENRHSIYFQVPSKTRQQEQIQY
O_anatinus      GLERR-RRYQRNRK-----VFPLFSSLKSVRPKKRSKPFLELRKPVLSRSFVSSGNS-GFILKLGK----RGVRSSGSSSLARISVNQEENEGKECNDSAVSKLSKYDQLAASVNLQELLQF
G_gallus        MIEGKNLKSLEKPKEFKERIFEESEHPSLLHEKH--QLYKPLQLPCIPRKSVQSLQTTRFSMLQTETGREEKENPMEQSVATSSSSSHAENPSQMVIGTATTTNLQVAQVPSIASFQSQTQF
P_sinensis      IIERK-SKNLEKRKEFKERTFEEIGNLHLLNGKH--QQYKPLHLPCMPQKLVQSVQTFRLSLQRTETGGEERDNSMDHSGIASISSSQVDNETQIFNESAGTNNPKIAEVPSLASLQNQTQF
S_harrisii      VLEEK-NKELEKTKECEGRIFEEHGHFRVLNENP--Q-SSQLFLPYNYQNLVRPVRNDRHFSEPGGTDK-KE-GSSSHSTLARIDIRPERKEILEFDVSVMN---KYSQVSPHKSTQNLMQF
M_musculus      ALDEK-NKNTEKRKEFEERIFEKDEQVSN-R-SS--Q-NSQLLLPCESQKFVRN--MN--SSEARVTDK-KEESSANQSKFVRSDVRQKENNPQIFNDSGMDSNSKSSHIPAVKSSQGFMQF
P_troglodytes   TLEEK-NKNTENRKELKERIFGKDEHVLTLN-KT--Q-SSQLFLPYESQKLVRPIKMH--SSEPRVADM-KEESSAKQSKLANIDFRQKENDTQIFNDSAVDNHSKCSHITTITSSQKFMQV
H_sapiens       TLEEK-NKNTENRKELKERIFGKDEHVLTLN-KT--Q-SSQLFLPYESQKLVRPIKMH--SSEPRVADI-KEESSAKQSKLANIDFRQKENDTQIFNDSAVDNHSKCSHITTITSSQKFMQV
                                    *                                                                                         :         .:      *   *

L_chalumnae     RQFATQKQTNLQQWLPSVTTLLGKTETNIAALELMYLSKDSVYASE----TESFAKAHEDYLENCAEQND--------QIFLPEPSEREEP---IHEKELCSTPTFKMN--HSIECEGSSGK
O_anatinus      RLNRKFHT----QLIS-----TQIKDAECQDENPKRAVNDSTCISHD-TSAENFGESKEDYPVAVYKNIEQFPCAPKSSSVGRNTEILRTSESTVMEGQLPLTSPFVLDQ--NIDSEGPNQD
G_gallus        RLLRPPKQVTTNQQFESEKSVIEDQEAKCDDKEAGDEPKDSSYIPQD---IRTYFKTNENNPDTAAEGAERFLRAPETPEFVGTPNSK------GKKSQFSKTPPFDFI--HNMGCEEATSK
P_sinensis      RLLVPQKQTACKQWYEKGN-----KDVECPDMGAASQSKDSAYISQQNVHTECFIKSSEDNPKAAEESTEHFPETPEMPLFIRTPESS------GKKAHFFKTPPFEFCRSHNLGSEGQTPK
S_harrisii      RLSCLEKQSNYKQTFDKGN-----TDSEYGKKETERELKDSAVLPQD-SHTEHMDKLVEVSHDETEENTEKFPSTPEMPPFLRTPEFLRTPEC-VEKLEFSKSPSSELLRNI--GFEGKTQK
M_musculus      RLNQPN----YNQRIEKEH-----IDAECGDKETVRQVRESKCSTQA-LYIEHFGKSIENNSVE-EERDENFPQTPETPSFLRTPEALKTPES-MEKMQFPKSPFFEITKNA--TSEGHKQK
P_troglodytes   RLLTPQKQSNSNQWSEKGD-----KDAEYGDKGTVRQVRESKCTSQA-IYTEHFGKSIENDSDEVEERAENFPRTSEIPIFLGTPKAVKAPES-LEKIKFPKTPPFEINRNRNAVPEVQTEK
H_sapiens       RLLTPQKQSNSNQWSEKGD-----KDAEYGDKGTVRQVRESKCTSQA-IYTEHFGKSVENDSDEVEERAENFPRTSEIPIFLGTPKAVKAPES-LEKIKFPKTPPFEINRNRNAVPEVQTEK
                *           *            : :          .:*    .     .   :  *       :  :          .     .           : .:  :    :        *  . .

L_chalumnae     -STPFSFLMASTPKTPDFNLFDSSVFGTVNTPNQLVVNYSSTNQDQANPQEETESTFDRAQGEEEFTFSFQTQRSPHPLRDKKDDFSFPFSFGQDPQMSQPSSFKGFQCSSQSAKQFTFF
O_anatinus      ASPTIPFFLTSATQSPGFSFFDSALFTG-DSPNQFAENYSAENFNRGSSQKDIGDLFGKLE-EDTFTFAFPTESSTHKFEGGKDDFSFSFPFESDQSLQPCSST----SSPQTTKQLIFF
G_gallus        -SPAFFSLLGITQKSPGFNLFDSSVFGAENSSDEIDESYSVGNLNPMSPHKDFGSLFGKSESEDTFAFPFPLESTSHAFGDGKDDFSFPFAFGQDQRSSQSPSVKGFHSSLQNTKPSTFF
P_sinensis      -SPAFSFLMACTQKSPGFNLFDSSVFGAENSSDQTDESYSAGNLNPVSPHKDIGSLFGKLENEDAFTFSFPSEPSSHTYGDGKDDFSFPFAFGQDQRSSHSSSLKGFHSSSQNTKPFTLF
S_harrisii      TPQAFSFLMG-SSISPGFNLFESSEFGNENLPDQFDENYSSGNLNPVSSQKDIGGLFGKLEGEDTFTFPFSSEPSTHTFGDGRDDFSFSFSFEQDQRSSHSSS-NSFP-SSHNAKQFTFF
M_musculus      DSPGFSFLMSYTSRSPGLNLFDSSVSDSEISSDQFNEHYSAVNLNPSSSQQGIGNLFGKSEGEDAFTFSFSSD-SSHTFGAGKDDFSFPFSFEQDPSTMTSSSSKDFSSS-QNKTQFMFF
P_troglodytes   ESPGLSFLMSYTSRSPGLNLFDSAVFDTEISSDQFNEHYSARNLNPLSSEQEIGNLFEKPEGEDGFTFSFPSDTSTHTFGAGKDDFSFPFSFGQGQNSIPSSSLKGFSSSSQNTTQFTFF
H_sapiens       ESPGLSFLMSYTSRSPGLNLFDSSVFDTEISSDQFNEHYSARNLNPLSSEQEIGNLLEKPEGEDGFTFSFPSDTSTHTFGAGKDDFSFPFSFGQGQNSIPSSSLKGFSSSSQNTTQFTFF
                    :  ::  :  :* :.:*:*:        ::    **  * :  . .:     : : : *: *:* *  : : *     :***** * * .         *      * :. .   :*
```

Supplementary Figure 1. Sequence alignment. Sequence alignment of SIX6OS1 homologues in vertebrates. Amino acid sequences of *H. sapiens* (human, Q8N1H7), *M. musculus* (mouse, NP_083381), *P. troglodytes* (Chimp, H2Q8E6), *S. charissii* (Tasmnaina devil, G3WQS7), *O. anatinus* (Latypus, F6ZZ02), *P. sinensis* (Chinese turtle, K7GAG2), *G. gallus* (Chick, E1C952) and *L. chalumnae* (West india coelacanth, M3XIB0) are derived from the UniProt database. Mouse data are derived from cDNA clone (*4930447C04Rik*). The protein is conserved among most vertebrates (with the exceptions of Amphibia, Reptilia and Actinopterygii). SIX6OS1 orthologues were identified by BLASTP and/or UniProt server. Phylogenetic analysis through genome databases indicated that *SIX6OS1* is a unique gene without paralogues that seems to appear firstly in the genomes of cartilaginous fish (absent in ray-finned fish) and can be clearly identified in the genomes of lobed fin fish (Sarcopterigii as coelacantus), Sauropsida (birds and turtles but not in lizards and amphibians) and mammals. When no orthologues were found deposited in databases (i.e. boney fish, and reptiles), we verified its presence/absence by intensive tBLASTN search against their genomic sequences. Amino acid alignments were performed with ClustalW, using the default settings. No hits were found against the recent sequenced genome of Spotted gar (*Lepisosteus oculatus*, unduplicated genome from the sister lineage of teleost named Holostei) when using as a probe the sequence of the west india coelacanth. However, a small piece of homology was found in the genome of the shark elephant (scaffold_114 from position 2479341 to 945433 at http://esharkgenome.imcb.a-star.edu.sg/blast/ or http://skatebase.org/ skateBLAST) covering the conserved AKEYFKKK sequence and flanking residues. This recent evolutionary origin of SIX6OS1 is in concordance with the evolutionary origins of SYCE1 and SYCE3 (billateria and vertebrates, respectively) and in contrast to the more ancestral origin of the proteins SYCE2, TEX12 and SYCP1-3[1,2]. The variant rs1254319 (p.Leu524Phe) is indicated (grey).

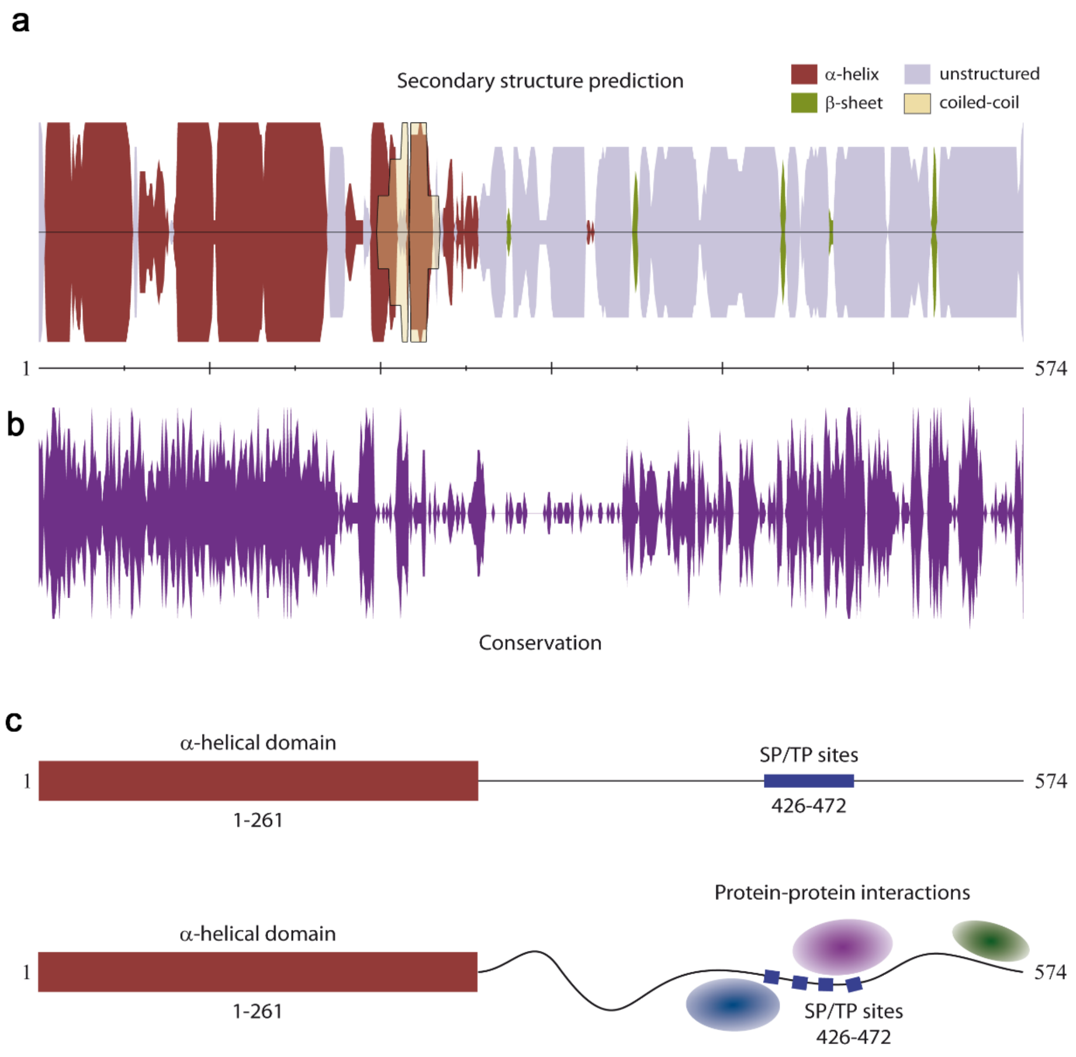

Supplementary Figure 2. Sequence analysis of SIX6OS1. (a) Secondary structure analysis of mouse SIX6OS1 in which the α-helical, β-sheet, unstructured and coiled-coil predictions are plotted on the basis of their confidence as calculated by JPred4[3]. (b) Conservation of the SIX6OS1 sequence, based on an alignment of all full length sequences produced in MUSCLE[4], plotted as the per residue conservation scores calculated in Jalview 2[5]. (c) Schematic diagram of the predicted SIX6OS1 structure, in which an N-terminal α-helical domain is linked to an unstructured protein-protein interaction module in the C-terminus through a flexible linker sequence. The putative protein-protein interaction region of the C-terminus includes multiple predicted phosphorylation sites, including four SP/TP potential CDK sites between amino acids 426-472, which may function in the dynamic regulation of interactions.

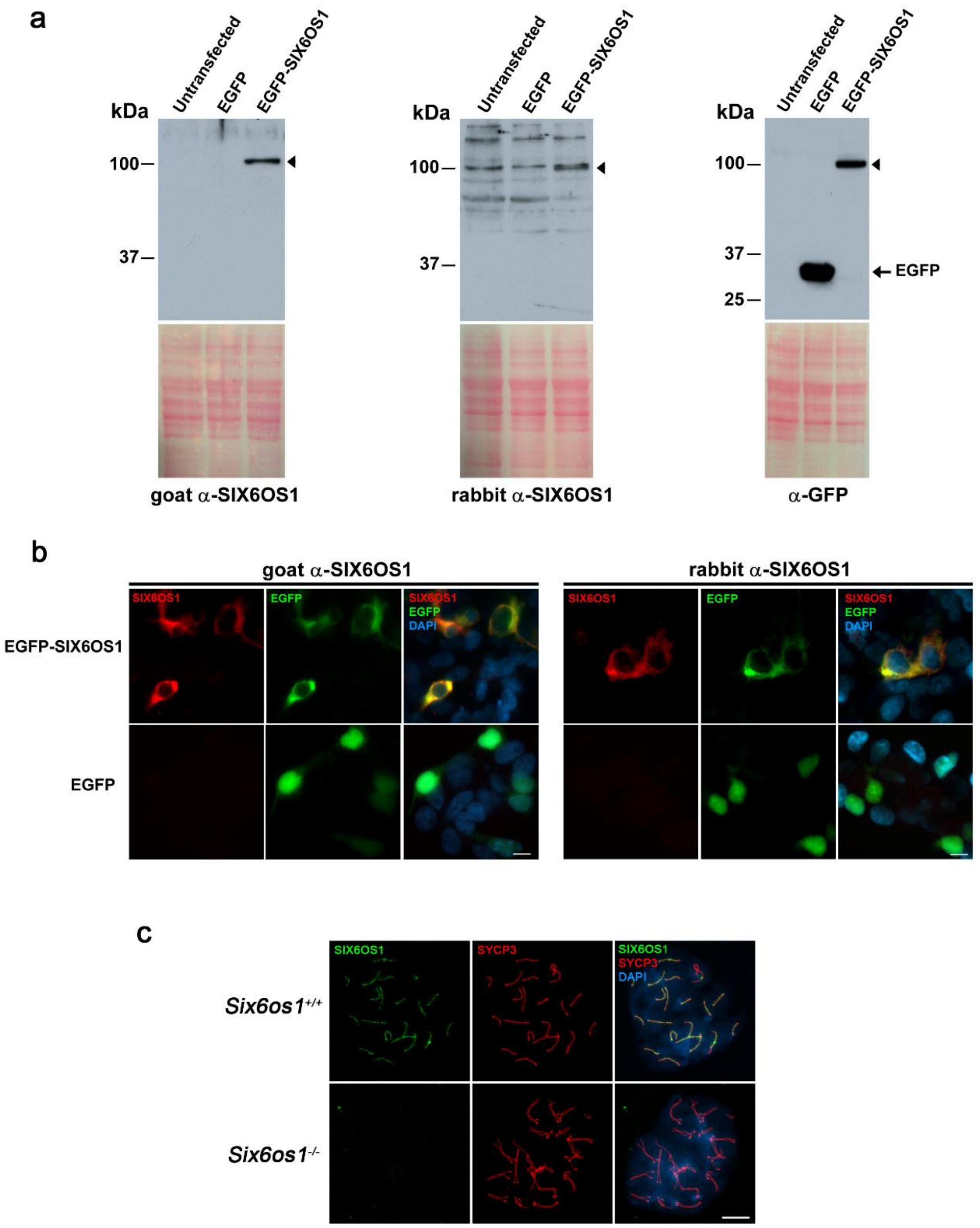

Supplementary Figure 3. Validation of SIX6OS1 antibodies.

(a) HEK 293T cells were transfected with a plasmid encoding GFP-SIX6OS1 or GFP and the whole extracts were analyzed by western blot using goat α-SIX6OS1 (left panel), rabbit α-SIX6OS1 (central panel) and α-GFP (right panel). Ponceau S staining of the blotted membranes was used as loading control. A band around 100 kDa, corresponding to the expected GFP-SIX6OS1 fusion protein (32,7 kDa + 70 kDa), was

detected with the goat α-SIX6OS1, rabbit α-SIX6OS1 and goat α-GFP (arrowheads). (b) Immunofluorescence of HEK 293T cells transfected with plasmids encoding GFP-SIX6OS1 or GFP. SIX6OS1 was detected with either goat α-SIX6OS1 (left panel) or rabbit α-SIX6OS1 (right panel, red) and GFP by direct fluorescence signal (green). Green and red signals co-localize in the cytoplasm of the transfected HEK 293T cells. (c) Double immunofluorescence of spermatocytes at pachytene stage obtained from *Six6os1*$^{+/+}$ and *Six6os1*$^{-/-}$ mice using the polyclonal rabbit antibody α-SIX6OS1 (green) and mouse α-SYCP3 (red). The experiments were reproduced three times. Bars represent 10 µm.

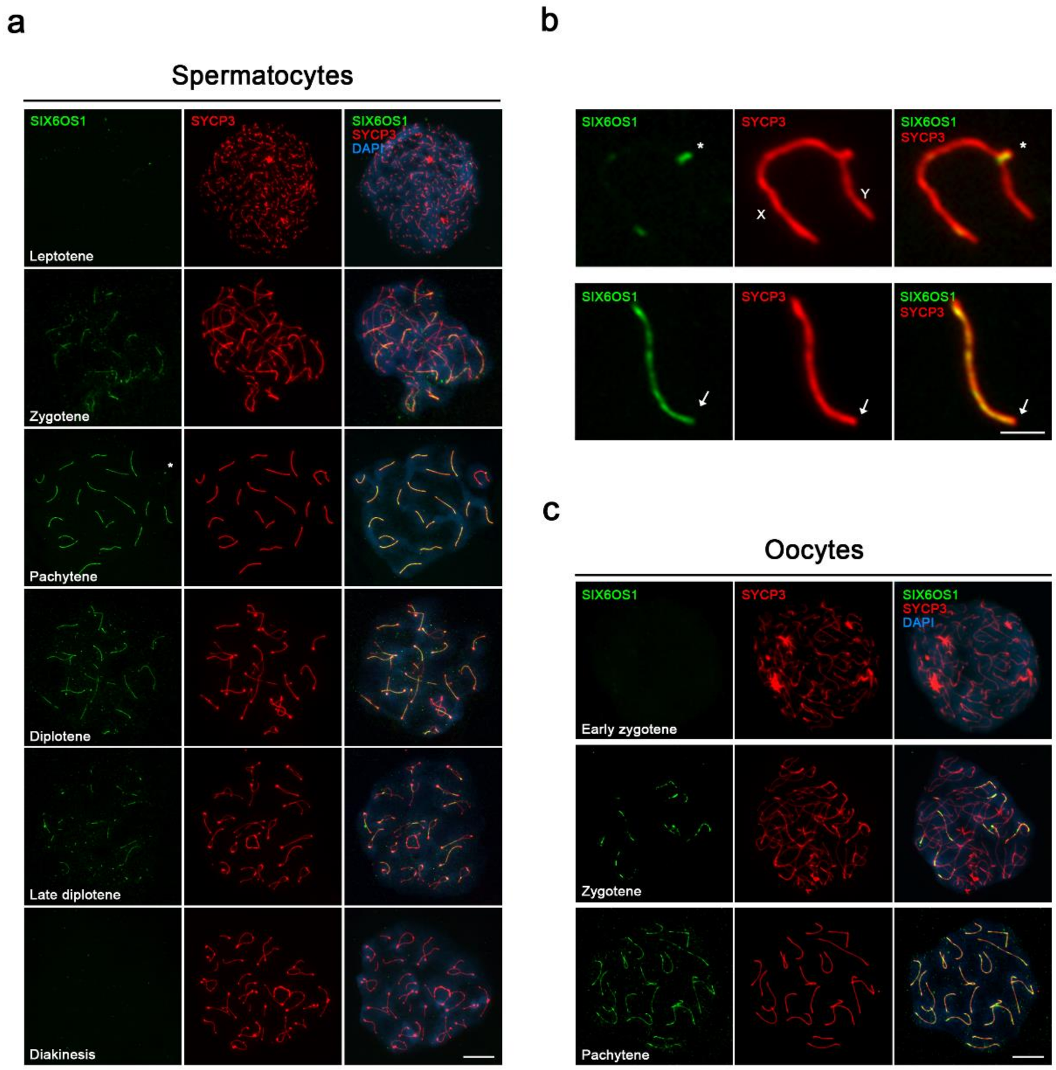

Supplementary Figure 4. Distribution of SIX6OS1 in mouse meiotic prophase I. Double immunolabelling of endogenous SIX6OS1 (green) and SYCP3 (red) in meiocytes. (a) In spermatocytes, SIX6OS1 is not present at leptotene, and appears in the synapsed regions of the lateral elements (LEs) at zygotene. During pachytene, SIX6OS1 is located at the synapsed autosomal LEs and at the pseudoautosomic region (PAR) of the sexual XY bivalent. At diplotena, SIX6OS1 appears on synapsed LEs but is absent from de-synapsed axial elements (AEs), with no signal when spermatocytes reach diakinesis. (b) Details of the sex chromosomes (upper panel) and an autosomal AE in pachytene (lower panel). Asterisks show SIX6OS1 signal in the PAR. The signal of SIX6OS1 is diminished at the telomeres (see arrows). (c) Distribution of SIX6OS1 in oocytes along synapsed AEs from zygotene to pachytene. The localization mimics that observed in males. Bar in panels a and c, 10 µm. Bar in panel b, 2 µm.

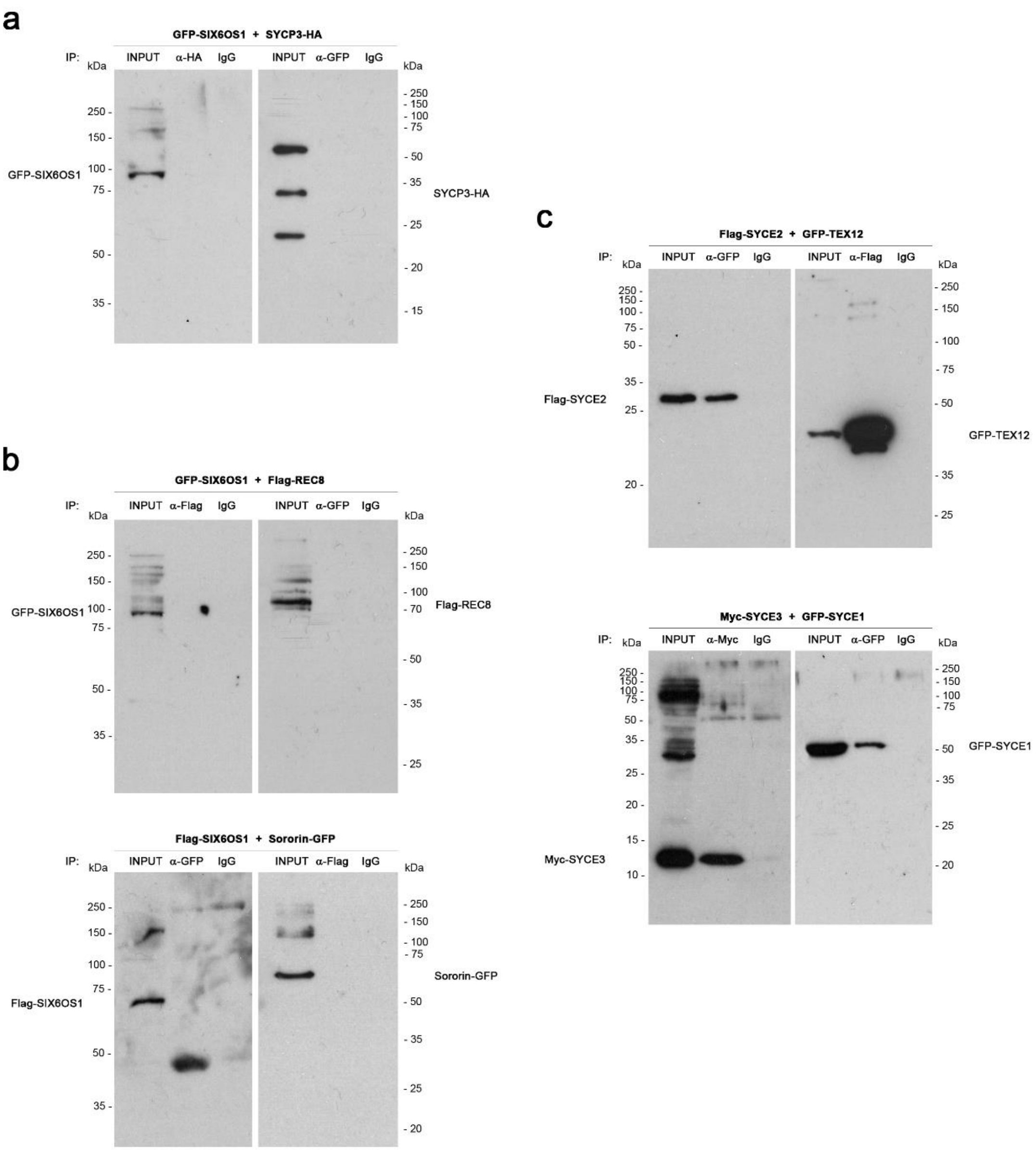

Supplementary Figure 5. SIX6OS1 does not interact with SYCP3, REC8 and Sororin. HEK 293T cells were co-transfected with the indicated expression vectors. Immunoprecipitations (IPs) were performed with the indicated antibody. (a) SIX6OS1 does not co-immunoprecipitate with either SYCP3, or the cohesins REC8 and Sororin (b). (c) IPs of TEX12 with SYCE2, and SYCE3 with SYCE1, were used as positive controls. The experiments were reproduced three times.

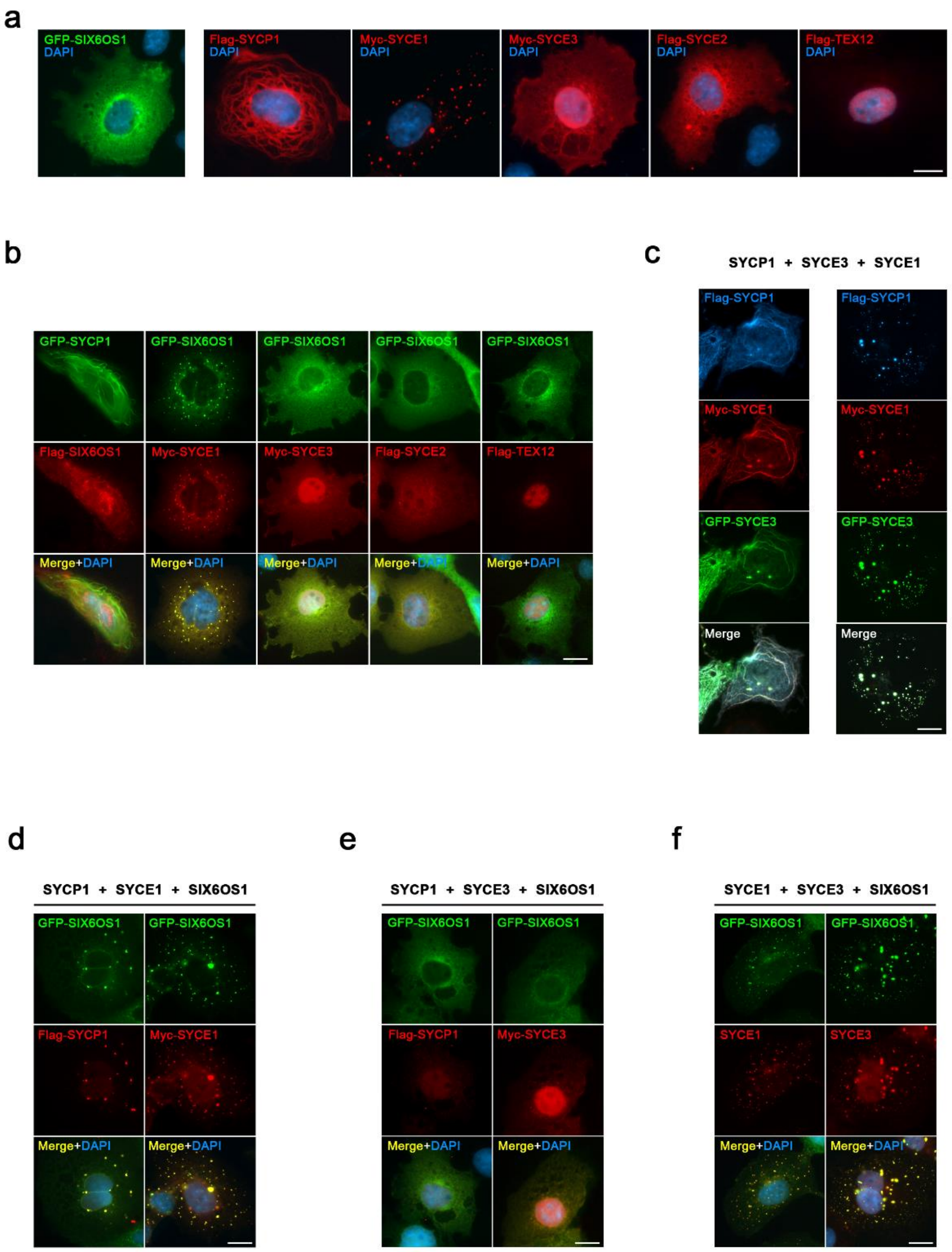

Supplementary Figure 6. Polycomplex formation by synaptonemal complex (SC) proteins in COS7 cells. COS7 cells were transfected with expression vectors encoding SIX6OS1, SYCP1, SYCE3, SYCE1, SYCE2 or TEX12 alone or in different combinations. (a) Individual transfections of all SC proteins. (b) Co-transfection of *Six6os1* with either *Sycp1*, *Syce1*, *Syce3*, *Syce2* or *Tex12*. SIX6OS1 localization only changes in the presence of SYCE1. (c) Co-transfection of Flag-*Sycp1*, Myc-*Syce1* and GFP-*Syce3* showing co-localization with two different patterns: polycomplexes (left) and cytoplasmic speckles (right). (d) Co-transfection of Flag-*Sycp1*, Myc-*Syce1* and

GFP- *Six6os1* showing co-localization in cytoplasmic speckles. (e) Co-transfection of Flag-*Sycp1*, Myc-*Syce3* and GFP-*Six6os1* showing no co-localization. (f) Co-transfection of *Syce1*, *Syce3* and GFP-*Six6os1* showing co-localization in cytoplasmic speckles of either SYCE1 (anti-SYCE1) with GFP-SIX6OS1 (anti-GFP) or between SYCE3 (anti-SYCE3) with GFP-SIX6OS1 (anti-GFP). The experiments were reproduced at least three times. Bar in panels, 15 μm.

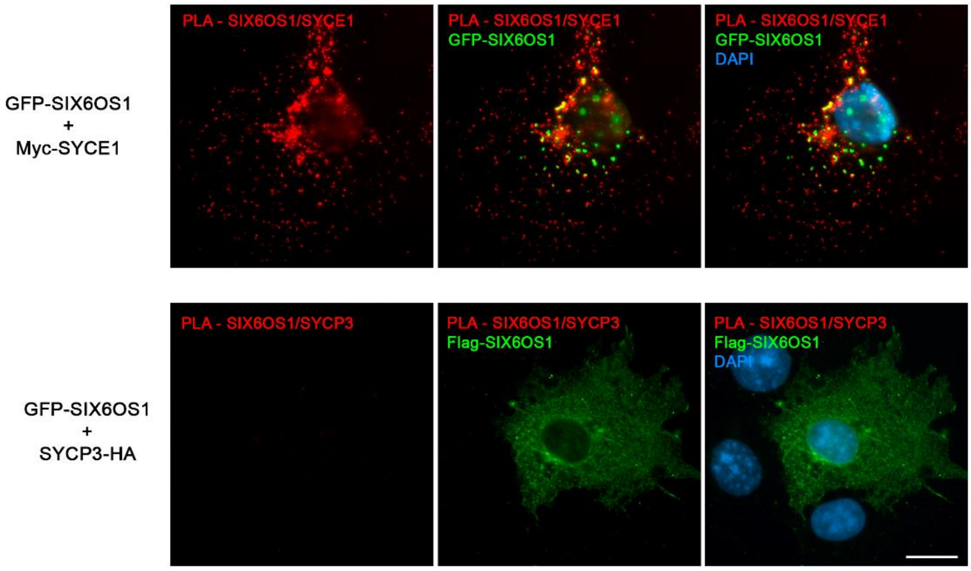

Supplementary Figure 7. Proximity ligation assay. COS7 cells were transfected with plasmids encoding EGFP-SIX6OS1 and Myc-SYCE1 (upper panel), and Flag-SIX6OS1 with SYCP3-HA (negative control, lower panel). Proximity Ligation Assay (PLA) was performed using goat αSIX6OS1 (sc-5385) and rabbit αSYCE1, with the corresponding anti-goat PLA Probe PLUS and anti-rabbit PLA probe MINUS. Red fluorescence indicates close proximity (interaction) between SIX6OS1 and SYCE1 at the cytoplasmic speckles where both proteins co-localize. Similarly, Proximity Ligation Assay was performed on the negative control (no interaction previously observed by IP, see Supplementary Fig. 5) using goat αSIX6OS1 and rabbit αSYCP3. No red labelling was observed. The experiments were reproduced twice. Bar represents 15 μm.

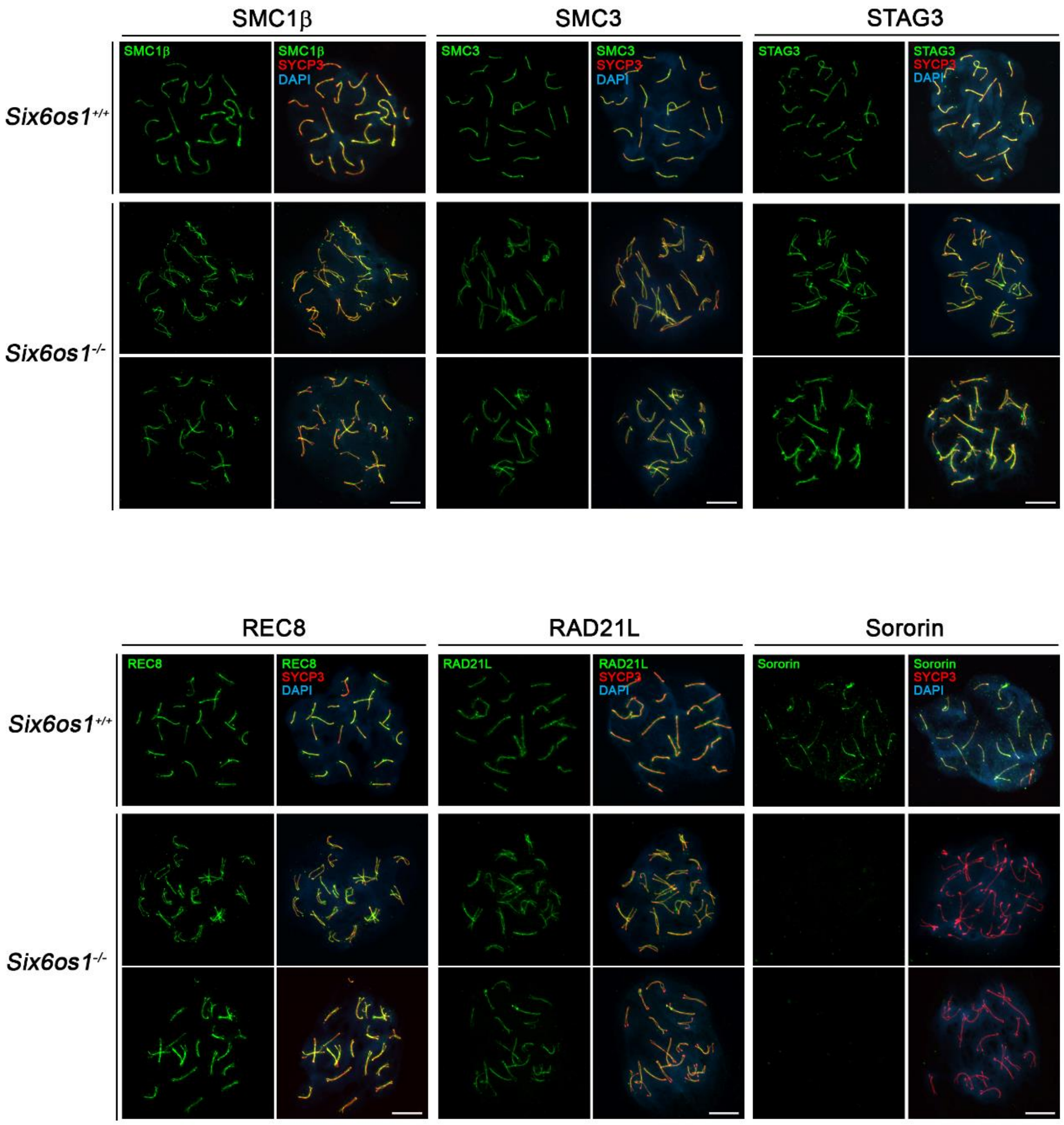

Supplementary Figure 8. **SIX6OS1 and cohesin loading**. Double immunofluorescence of SYCP3 (red) with either SMC3, SMC1β, STAG3, REC8, RAD21L or Sororin (green) in wild-type and *Six6os1*$^{-/-}$spermatocytes. In wild-type pachytene spermatocytes, the cohesins SMC1β, SMC3, STAG3, REC8 and RAD21L colocalize with SYCP3 along the autosomal AEs and sex AEs, whereas Sororin co-localizes to synapsed LEs and the pseudoautosomal synapsed region of the XY bivalent. In the absence of synapsis in *Six6os1*$^{-/-}$spermatocytes, the levels or distribution of cohesin subunits SMC1β, SMC3, STAG3, REC8 and RAD21L are not altered, whereas Sororin is not loaded, as expected for a cohesin located at the central element (CE) of the SC. Bar in panels, 10 μm.

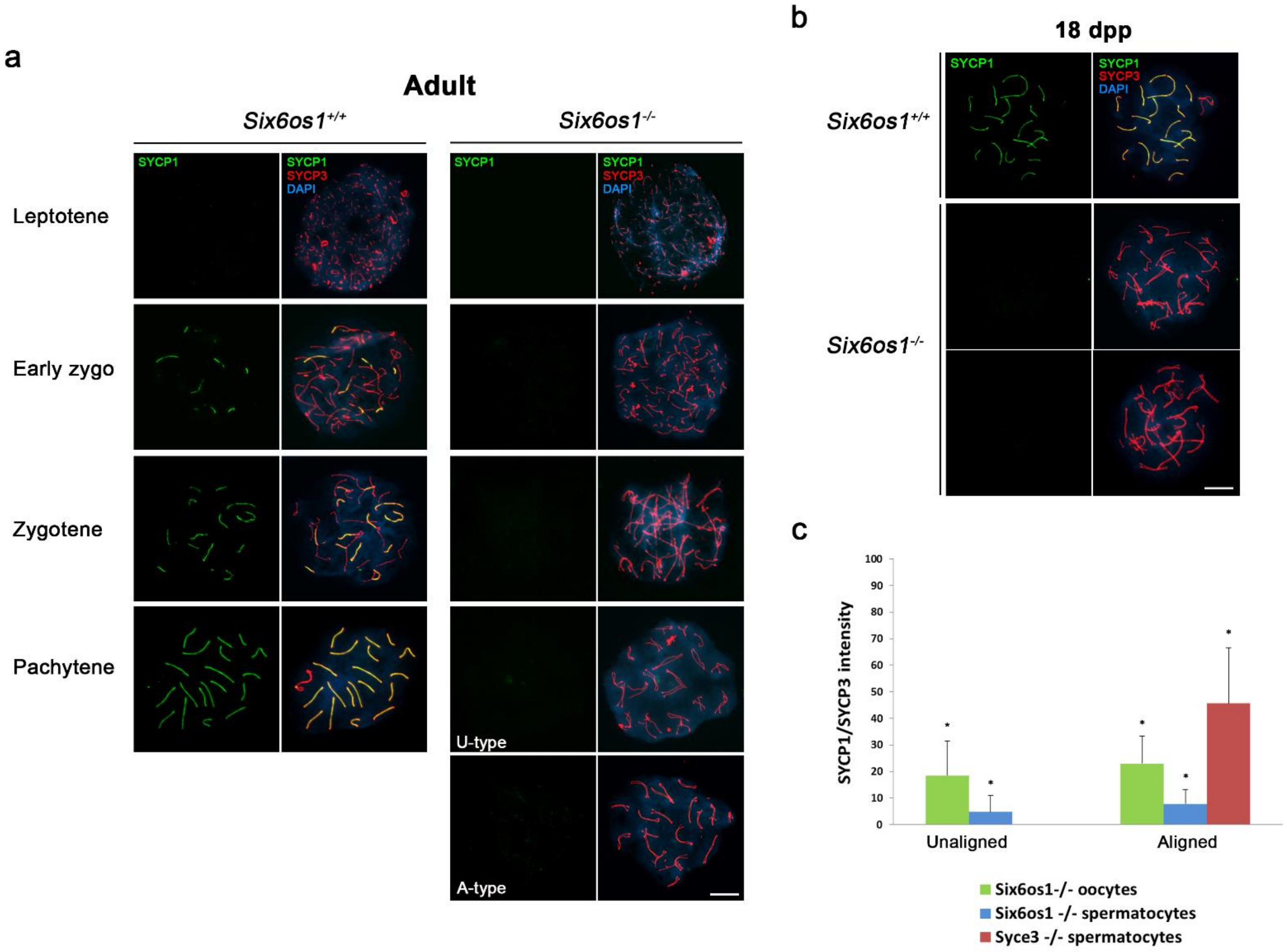

Supplementary Figure 9. SIX6OS1 is necessary for the initiation of synapsis. Double labelling of SYCP3 (red) and SYCP1 (green) in adult (a) and 18 dpp (b) mice. (a) *Six6os1*$^{-/-}$ adult spermatocytes assemble AEs of normal morphology and composition (SYCP3) from leptotene to zygotene. They arrest at pachytene-like stage, showing two phenotypes, A-type, with aligned AEs and U-type, poorly aligned or even completely unaligned. In the absence of SIX6OS1, SYCP1 is unable to load to the AEs or appears in very low levels. (b) *Six6os1*$^{-/-}$ spermatocytes at the first wave of spermatogenesis (18 dpp) fail to synapse, mimicking the adult phenotype, with a complete absence of SYCP1. (c) Quantification of SYCP1 levels (relative to SYCP3 fluorescence intensity) at pachytene-like stage of *Six6os1*$^{-/-}$ spermatocytes and oocytes, and *Syce3*$^{-/-}$ spermatocytes. Represented data are related to wild-type pachytene levels, considered as 100%. Welch´s *t*-test analysis: * $p<0.0001$; n= 30 AE/LEs, mean ± s.d. Bar in panels, 10 µm.

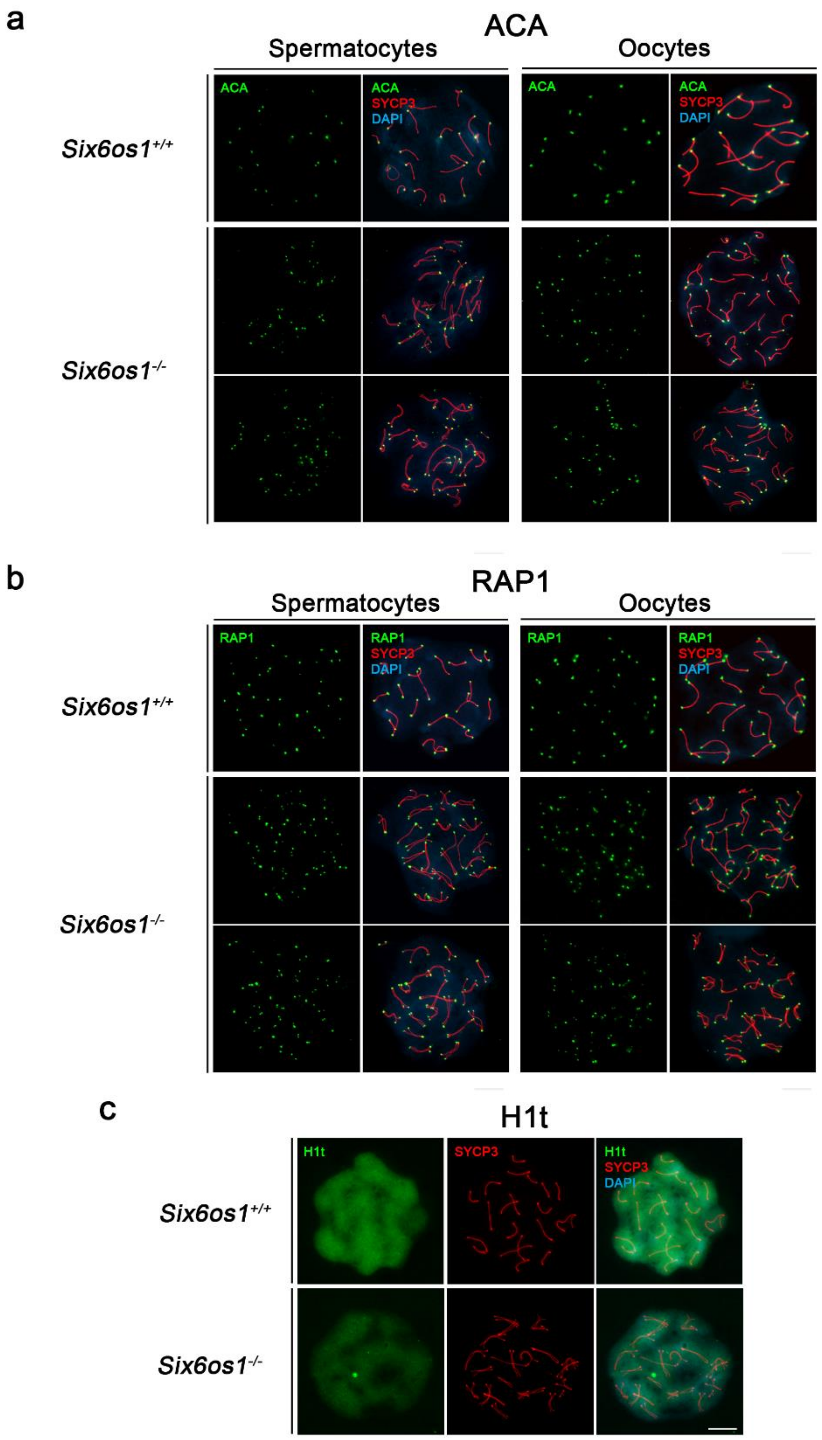

Supplementary Figure 10. Centromeres and telomeres fail to synapse in *Six6os1*$^{-/-}$ meiocytes. (a) Double immunofluorescence of ACA (green) and SYCP3 (red) in spermatocyte (left panel) and oocyte (right panel) spreads. Wild-type pachytene spermatocytes show 21 single signals of ACA at one end of the LEs. However, the number of ACA signals is 40 in *Six6os1*$^{-/-}$ pachytene-like arrested spermatocytes owing

to the absence of synapsis. *Six6os1*$^{-/-}$ oocytes show 40 ACA signals vs 20 in wild-type. (b) Co-labelling of RAP1 (green) and SYCP3 (red) in spermatocyte (left panel) and oocyte (right panel) spreads. *Six6os1*$^{-/-}$ meiocytes show 80 RAP1 foci, one at each end (telomere) of the AEs, while wild-type meiocytes show 40 RAP1 signals. (c) Double immunofluorescence of H1t (green) and SYCP3 (red) in spermatocytes from *Six6os1*$^{-/-}$ and *Six6os1*$^{+/+}$ showing loading of H1t in the arrested pachytene-like and wild-type pachytene spermatocytes. Bar in panels, 10 µm.

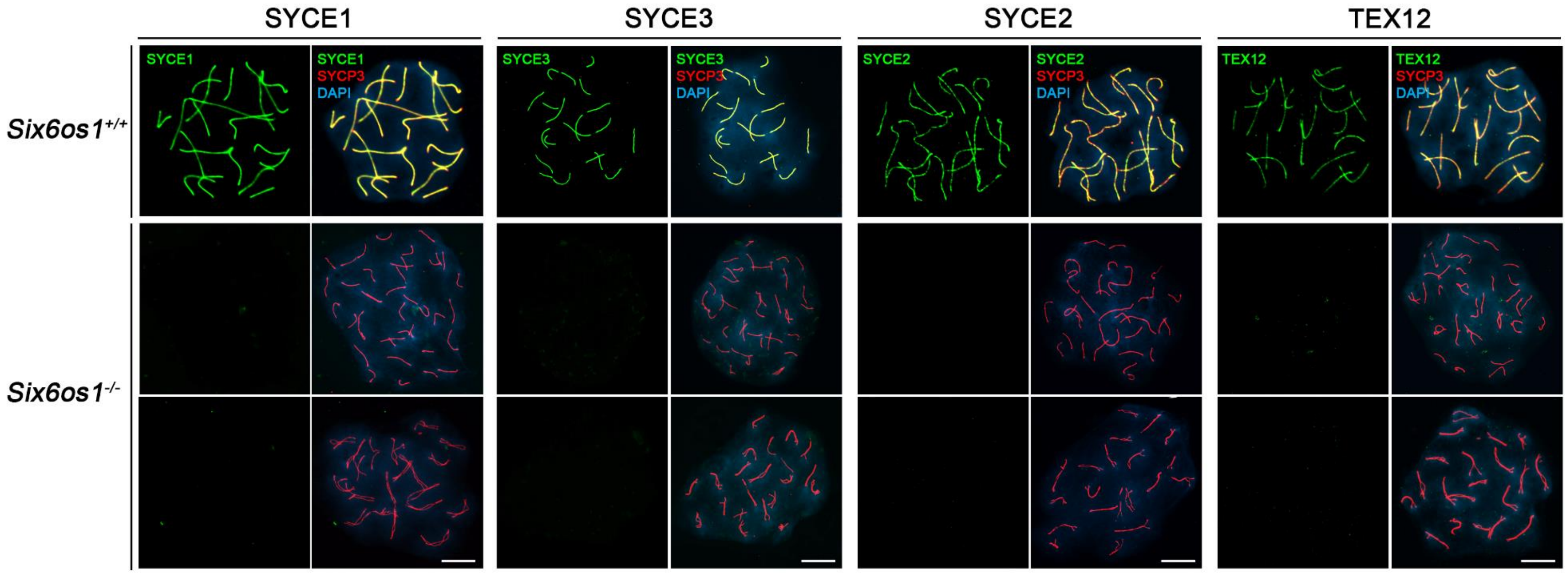

Supplementary Figure 11. ***Six6os1**$^{-/-}$* **oocytes lack CE proteins.** Double labelling of spreads of wild-type pachytene and *Six6os1*$^{-/-}$ pachytene-like oocytes with SYCP3 (red) and SYCE1, SYCE3, SYCE2 or TEX12 (green). All proteins are completely absent from AEs in *Six6os1* deficient mice. Bar in panels, 10 μm.

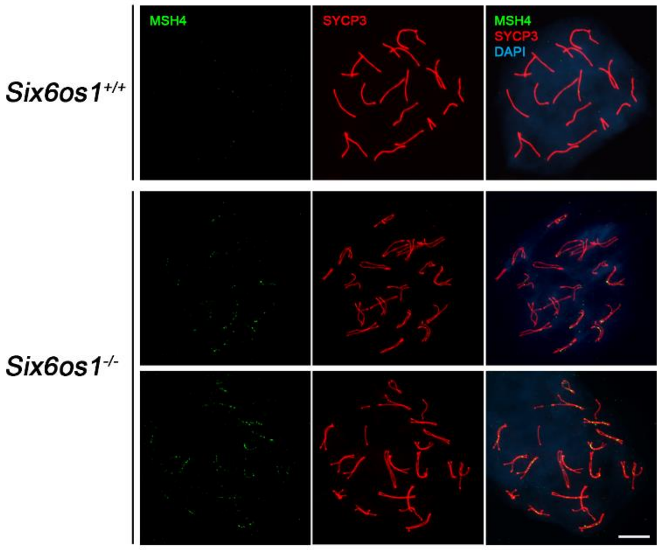

Supplementary Figure 12. Immunolabelling of MSH4 in the absence of SIX6OS1. Double immunolabelling of SYCP3 (red) with MSH4 (green) in wild-type pachytene and *Six6os1*$^{-/-}$ pachytene-like spermatocytes, showing that MSH4 persists in *Six6os1*$^{-/-}$ in contrast to wild-type. Bar in panels, 10 µm.

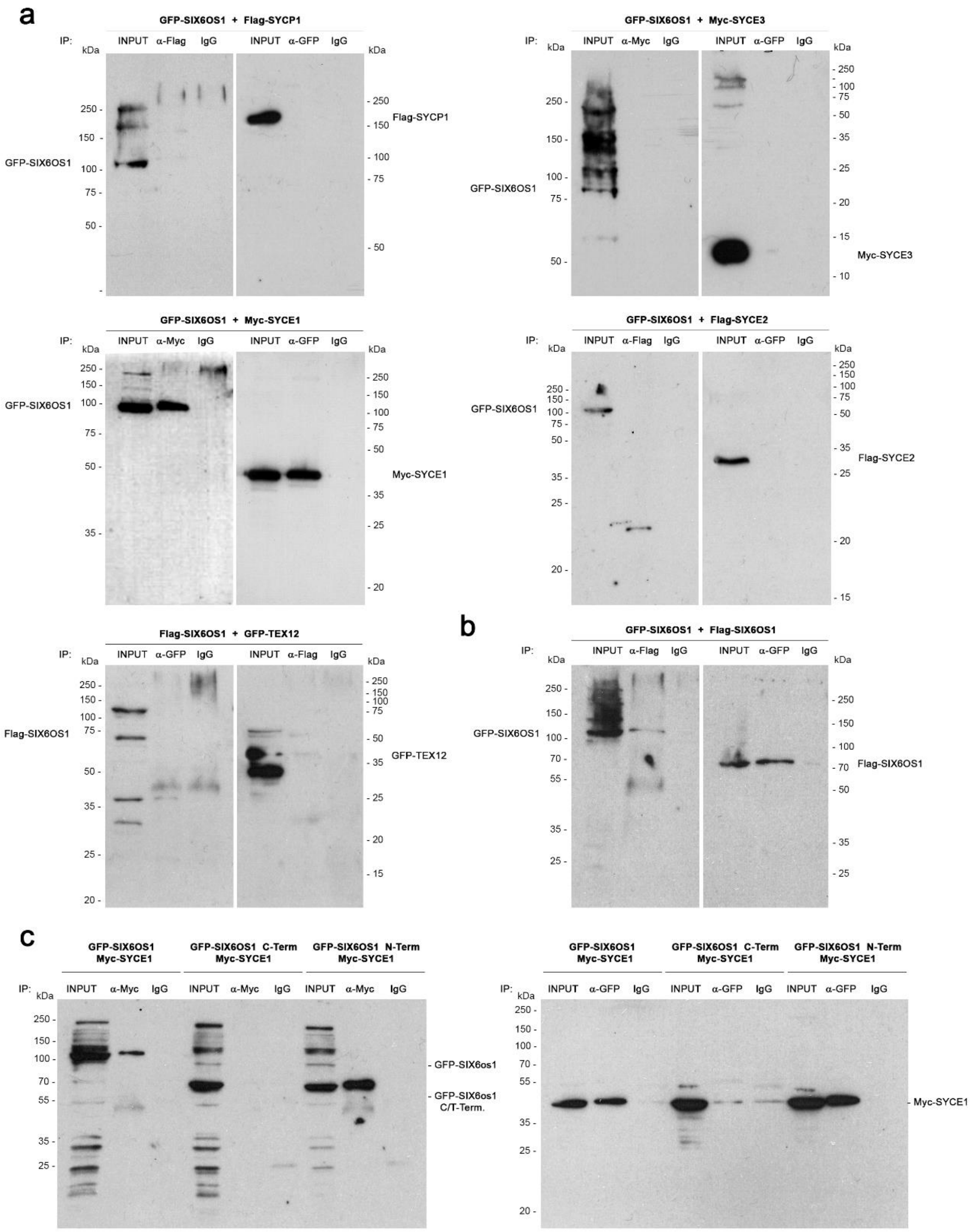

Supplementary Figure 13. Uncropped western blots of SIX6OS1 interactions. (a-c) HEK 293T cells were transfected or co-transfected with the indicated expression vectors. Protein complexes were immunoprecipitated overnight with either an anti-Flag, an anti EGFP or an anti-Myc antibody and were analyzed by immunoblotting with the indicated antibody. (a) SIX6OS1 co-immunoprecipitates with SYCE1 (as well as in the reciprocal IP) but not with either SYCP1, SYCE3, SYCE2 or TEX12. (b) SIX6OS1-

Flag co-immunoprecipitates with SIX6OS1-GFP, suggesting that it is able to form at least dimers. (c) SYCE1 co-immunoprecipitates with the SIX6OS1 N-terminal half (1-286) but not with the C-terminal half (287-574). IP of SYCE1 and full length SIX6OS1 was used as positive control.

Supplementary Table 1. Quantification of γ-H2AX levels and RAD51 foci in early meiotic prophase of spermatocytes.

γ-H2AX		Mean (intensity)	SD	n
Leptotene	WT	58,91	14,78	28
	KO	51,26	19,41	27
Zygotene	WT	55,00	13,91	36
	KO	57,20	16,75	30

RAD51		Mean (foci)	SD	n
Leptotene	WT	100,1	32,77	25
	KO	117,2	21,79	29
Zygotene	WT	86,73	24,38	30
	KO	130,2***	24,63	27

Welch´s *t*-test analysis between WT and KO: *** $p<0.0001$.

Supplementary Table 2. Quantification of γ-H2AX levels, and RAD51 and RPA foci.

γ-H2AX		Mean (intensity)	SD	n
Spermatocytes	WT	23,40	3,201	45
	A-type	30,74	3,593	39
	U-type	26,02	4,956	29
Oocytes	WT	20,85	3,48	27
	A-type	27,01	10,88	16
	U-type	28,14	9,830	27

RAD51		Mean (foci)	SD	n
Spermatocytes	WT	21,13	12,23	40
	A-type	121,7	21,85	32
	U-type	50,36	16,96	25
Oocytes	WT	21,90	12,11	20
	A-type	157,1	34,05	14
	U-type	147,8	33,96	10

RPA		Mean (foci)	SD	n
Spermatocytes	WT	10,38	9,485	29
	A-type	123,9	17,26	29
	U-type	85,80	15,83	20
Oocytes	WT	38,10	22,88	21
	A-type	158,2	44,52	10
	U-type	130,9	25,39	13

Significance of the comparisons between groups is shown in the plots of Fig. 8.

Supplementary References

1. Fraune, J., *et al.* Hydra meiosis reveals unexpected conservation of structural synaptonemal complex proteins across metazoans. *Proceedings of the National Academy of Sciences of the United States of America* **109**, 16588-16593 (2012).
2. Fraune, J., Brochier-Armanet, C., Alsheimer, M. & Benavente, R. Phylogenies of central element proteins reveal the dynamic evolutionary history of the mammalian synaptonemal complex: ancient and recent components. *Genetics* **195**, 781-793 (2013).
3. Drozdetskiy, A., Cole, C., Procter, J. & Barton, G.J. JPred4: a protein secondary structure prediction server. *Nucleic acids research* **43**, W389-394 (2015).
4. Edgar, R.C. MUSCLE: multiple sequence alignment with high accuracy and high throughput. *Nucleic acids research* **32**, 1792-1797 (2004).
5. Waterhouse, A.M., Procter, J.B., Martin, D.M., Clamp, M. & Barton, G.J. Jalview Version 2--a multiple sequence alignment editor and analysis workbench. *Bioinformatics* **25**, 1189-1191 (2009).
